# Metabolome Profiling of Heat Priming Effects, Senescence, and Acclimation of Bread Wheat Induced by High Temperatures at Different Growth Stages

**DOI:** 10.3390/ijms222313139

**Published:** 2021-12-05

**Authors:** Sachiko Matsunaga, Yuji Yamasaki, Ryosuke Mega, Yusuke Toda, Kinya Akashi, Hisashi Tsujimoto

**Affiliations:** 1 United Graduate School of Agricultural Sciences, Tottori University, 4-101 Koyama-Cho Minami, Tottori 680-8553, Japan; smatsunaga3005.research@gmail.com; 2 Arid Land Research Center, Tottori University, 1390 Hamasaka, Tottori 680-0001, Japan; yujyamas@tottori-u.ac.jp; 3 Graduate School of Science & Technology for Innovation, Yamaguchi University, 1677-1 Yoshida, Yamaguchi 753-8515, Japan; mega@yamaguchi-u.ac.jp; 4 Department of Agricultural and Environmental Biology, Graduate School of Agricultural and Life Sciences, The University of Tokyo, 1-1-1 Yayoi, Bunkyo, Tokyo 113-8657, Japan; yusuke0320.research@gmail.com; 5 Faculty of Agricultural Sciences, Tottori University, 4-101 Koyama-Cho Minami, Tottori 680-8553, Japan; akashi.kinya@tottori-u.ac.jp

**Keywords:** heat, metabolites, wheat, heat priming, acclimatization, senescence, molecular marker, arid region

## Abstract

Our previous study described stage-specific responses of ‘Norin 61’ bread wheat to high temperatures from seedling to tillering (GS1), tillering to flowering (GS2), flowering to full maturity stage (GS3), and seedling to full maturity stage (GS1–3). The grain development phase lengthened in GS1 plants; source tissue decreased in GS2 plants; rapid senescence occurred in GS3 plants; all these effects occurred in GS1–3 plants. The present study quantified 69 flag leaf metabolites during early grain development to reveal the effects of stage-specific high-temperature stress and identify markers that predict grain weight. Heat stresses during GS2 and GS3 showed the largest shifts in metabolite contents compared with the control, followed by GS1–3 and GS1. The GS3 plants accumulated nucleosides related to the nucleotide salvage pathway, beta-alanine, and serotonin. Accumulation of these compounds in GS1 plants was significantly lower than in the control, suggesting that the reduction related to the high-temperature priming effect observed in the phenotype (i.e., inhibition of senescence). The GS2 plants accumulated a large quantity of free amino acids, indicating residual effects of the previous high-temperature treatment and recovery from stress. However, levels in GS1–3 plants tended to be close to those in the control, indicating an acclimation response. Beta-alanine, serotonin, tryptophan, proline, and putrescine are potential molecular markers that predict grain weight due to their correlation with agronomic traits.

## 1. Introduction

Plants respond to abiotic stresses by increasing DNA transcription, resulting in increased RNA transcript levels of the responsive genes and accumulation of proteins and other metabolites. These molecular dynamics lead to visible phenotypic changes. Metabolite analysis quantifies the amounts of small-molecular-weight compounds such as organic acids, amino acids, and nucleic acids. The results reveal changes in their biosynthesis and breakdown in metabolic pathways in plants under different conditions. For example, drought stress induces proline and abscisic acid (ABA) accumulation which is regarded as targeted markers for drought response [1], whereas, heat stress does not induce such metabolites. Here, we conducted a comprehensive analysis of metabolite changes in wheat after heat exposure using metabolomic analysis.

Metabolomic analysis describes overall change in stress-responsive metabolites and may therefore explain plant phenotypic changes based on the kinds and the quantities of the metabolites, since metabolites are the functional molecules that control changes in the phenotype [2]. This method can monitor transient responses to the environment during growth and can reveal markers that indicate or quantify responses to environmental factors during specific growth phases. A complete picture of changes in the metabolome will elucidate the metabolic pathways that regulate adaptability to the environment. Thus, metabolome analysis can contribute to more accurate selection of stress-tolerant genotypes in crop breeding; this approach has been applied for various environmental stresses, such as drought [3], high temperatures [4,5], heavy metals [6,7,8], alkali compounds [9], low nitrogen [10], and low phosphorus [11].

In our previous study, we exposed the bread wheat cultivar ‘Norin 61’ to high temperatures (38 °C), which is the temperature in Wad Medani, Sudan, the world hottest area for wheat production, during different growth stages, and analyzed the morphological and physiological stress responses [12]. Additionally, previous research reported 50% decrease in rubisco activity at temperatures near 38 degrees Celsius [13]. High temperature during the seedling and vegetative stages decreased plant height and the number of grains, whereas this stress during the reproductive stage significantly shortened the seed formation period and reduced grain weight. However, interestingly, high temperature during the seedling stage delayed senescence and extended the starch deposition period, therefore increasing the harvest index and thousand-kernel weight rather than reducing yield. We called the response to this treatment during the seedling stage the “heat priming” effect. The plants exposed to high temperature continuously from seedling to maturity showed delayed senescence compared with those exposed to high temperature only at maturity. We called this phenomenon “acclimation” to maintain photosynthetic activity longer. However, metabolites behind these growth-specific phenomena caused by high temperature remain to be identified. Here, we investigated the dynamics of 69 metabolites of the plants exposed to high temperature at different growth stages and found the metabolites that were most strongly correlated with agronomic traits. Our final goal is to isolate biomarkers for use in wheat breeding.

## 2. Results

### 2.1. Analysis of Metabolite Trends under Different High-Temperature Timings

**Figure 1:**
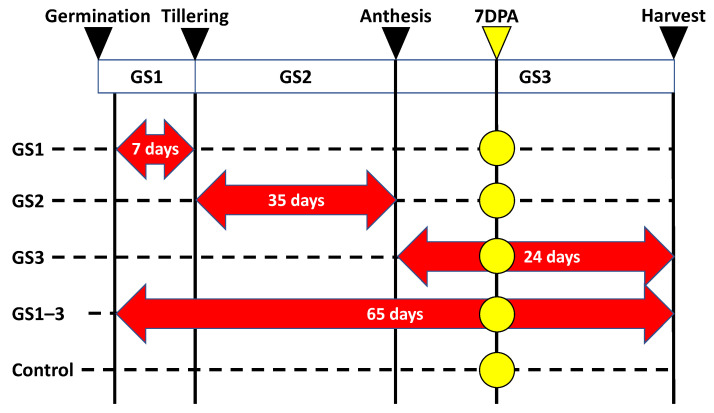
Timing of the heat treatment (red arrows) and metabolite sampling (yellow circles). Wheat plants were kept at 22 °C day/18 °C night (Control) or exposed to transient high temperature (Heat) at 38/18 °C during the indicated growth stages. Red arrows indicate the high-temperature treatment period with treatment days, and dashed lines indicate the period with normal temperature. GS, growth stages (1 = heating from germination to tillering; 2 = heating from tillering to flowering; and 3 = heating from flowering to full maturity); DPA, days post-anthesis.

Previous studies suggested that high temperature during certain growth stages affects the senescence rate during seed formation [12]. Therefore, to explore the metabolic changes during the seed formation period under each temperature condition (Figure 1), we quantified the stably detected metabolites (amino acids, organic acids, nucleic acids, and hormones) in the most recent flag leaves at 7 days post-anthesis (DPA) (Table S1).

**Figure 2:**
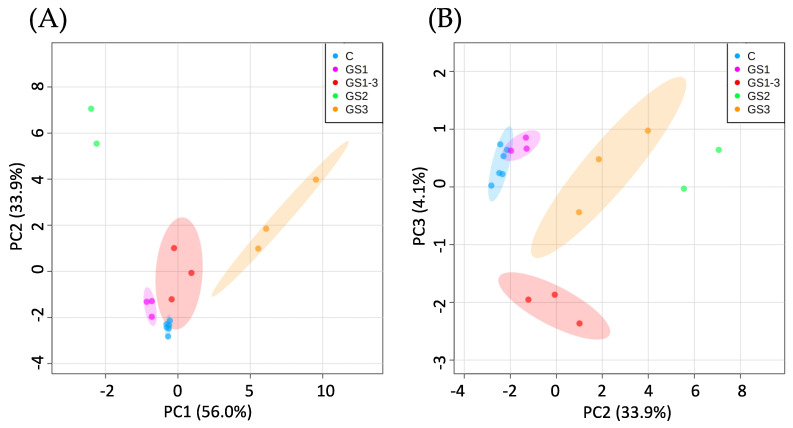
Results of the principal component analysis for the different temperature treatments for (**A**) principal components PC1 and PC2 and (**B**) principal components PC2 and PC3. GS represents the growth stages when the high-temperature treatment was applied (C = control, with normal temperatures; 1 = heating from germination to tillering; 2 = heating from tillering to flowering; and 3 = heating from flowering to full maturity; 1–3 = all stages). Stable 69 metabolites were used to perform PCA. The 95% confidence intervals are highlighted by the respective background color. GS2 could not be highlighted because the sample size is less than three replications.

All replicates of each condition were distributed close to each other in the principal component analysis (PCA) plot of the first two principal components, PC1 and PC2, which accounted for a high proportion (89.9%) of the variance (Figure 2A). The GS2 and GS3 samples were distributed far from the control samples, whereas the GS1 samples were distributed close to the control samples in PC1. For both PC1 and PC2, the GS1–3 samples, which were treated at high temperature during all growth stages, showed a distribution closer to the control than the samples treated only in GS2 or GS3. However, for PC3 (which explained only 4.1% of the variance) the values for GS1–3 samples differed from those for control and GS2, GS3 samples (Figure 2B).

### 2.2. Clustering and Heat Map Based on the Metabolite Response in Each High-Temperature Treatment

**Figure 3:**
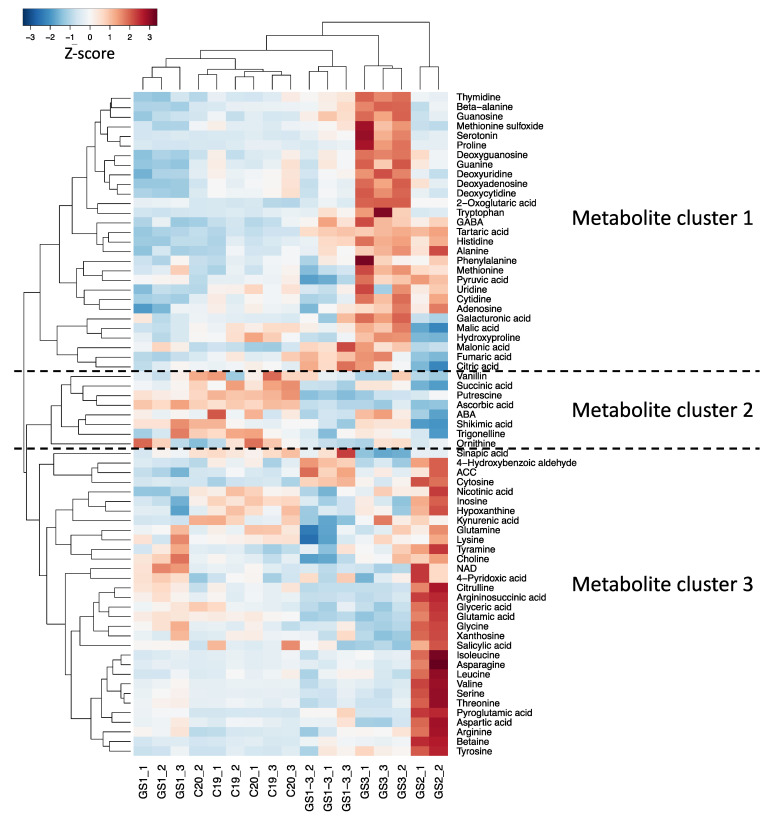
Hierarchical clustering and heat map for the results of high-temperature treatment during different growth stages: C = control, with normal temperatures; GS1 = heating from germination to tillering; GS2 = heating from tillering to flowering; GS3 = heating from flowering to full maturity; and GS1–3 = heating during all growth stages. Colors show increases (red) or decreases (blue) in metabolite levels in Z-score. Abbreviations: ABA, abscisic acid; ACC, 1-Aminocyclopropane-1-carboxylic acid; GABA, gamma-aminobutyric acid; NAD, nicotinamide adenine dinucleotide.

Clustering based on the quantified metabolite contents revealed three major clusters (Figure 3). In the GS1 samples, the levels of most metabolites were lower than in the control samples, especially in metabolite cluster 1. Metabolite cluster 1 contained many nucleic acids such as deoxyuridine, deoxycytidine, and adenosine, and amino acids such as tryptophan, proline, and phenylalanine. Metabolite cluster 2 contained ascorbic acid, putrescine, and ABA. Metabolite cluster 3 contained more branched-chain amino acids synthesized from pyruvate and amino acids, such as glycine and serine, synthesized from glycerate triphosphate.

### 2.3. Metabolites That Differed Significantly from the Control and Growth Stage-Specific Behavior

**Figure 4:**
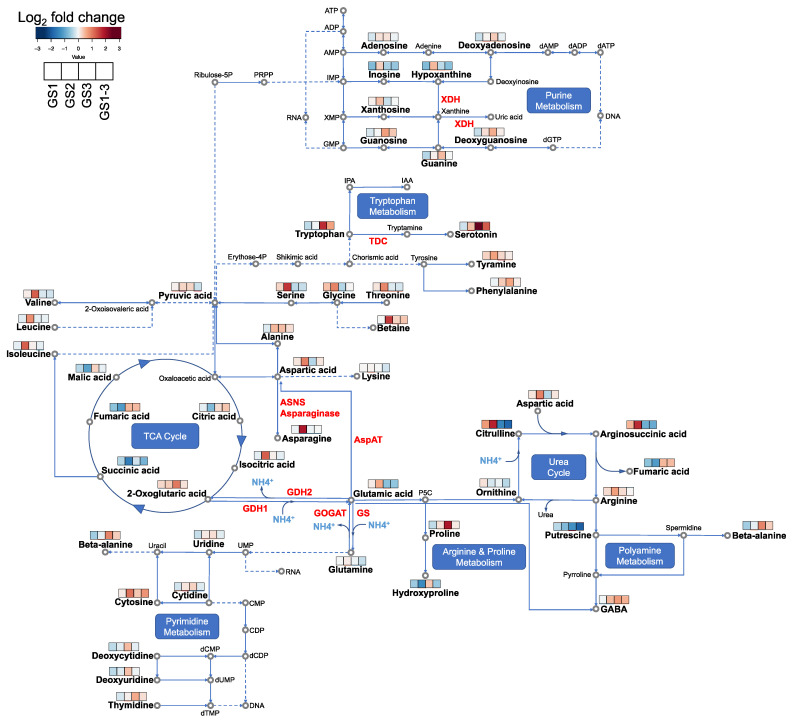
Changes in the metabolite contents of the flag leaves at 7 days post-anthesis under each condition: C = control, with normal temperatures; GS1 = heating from germination to tillering; GS2 = heating from tillering to flowering; GS3 = heating from flowering to full maturity; and GS1–3 = heating during all growth stages. The proposed metabolic pathways are based on the KEGG database (https://www.genome.jp/kegg/ accessed on 15 October 2021). The bars with four cells represent heat treatment during the four growth stages (GS1 to GS1–3, respectively); colors in the cells represent log_2_ of the change relative to the control. Abbreviations: XDH, xanthine dehydrogenase/oxidase; TDC, tryptophan decarboxylase; ASNS, asparagine synthetase; AspAT, aspartate aminotransferase; GDH, glutamic acid dehydrogenase; GOGAT, glutamine oxoglutarate aminotransferase; GS, glutamine synthase; GABA, gamma-aminobutyric acid.

**Figure 5:**
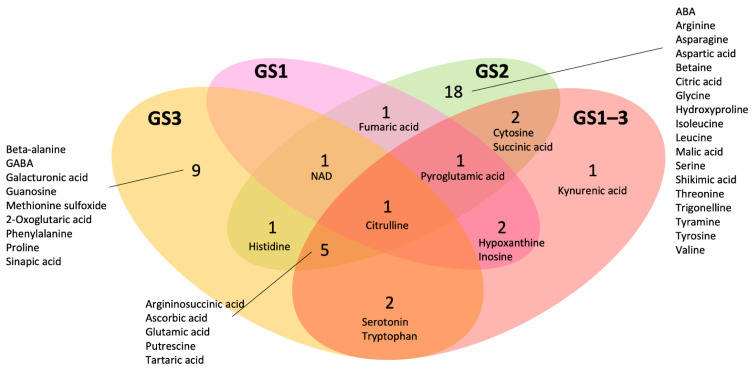
Venn diagram using the metabolites that significantly decreased to less than 0.67 times the control value or significantly increased to more than 1.5 times the control value in the heat-stressed plants. Growth stages: GS1 = heating from germination to tillering; GS2 = heating from tillering to flowering; GS3 = heating from flowering to full maturity; and GS1–3 = heating during all growth stages. Values represent the number of metabolites. Abbreviations: ABA, abscisic acid; GABA, gamma-aminobutyric acid.

In the GS1, GS2, GS3, and GS1–3 samples, we found 6, 30, 19, and 14 metabolites, respectively, that significantly (*p* < 0.05) decreased (less than two-thirds) or increased (more than one and a half fold) compared to the control (Table 1, Table S2, Figure 4 and Figure 5). Samples treated at high temperature only during GS2 accumulated the most stage-specific metabolites (18 metabolites). The metabolites that increased most (as a multiple of the control value) in GS2 were asparagine, followed by citrulline, argininosuccinic acid, serine, betaine, valine, isoleucine, nicotinamide adenine dinucleotide (NAD), tartaric acid, cytosine, aspartic acid, glycine, tyrosine, threonine, leucine, arginine, and tyramine. On the other hand, the metabolites that decreased most compared to the control were shikimic acid, followed by 4-hydroxyproline, malic acid, trigonelline, citric acid, and ABA (Table 1, Figure 5). The samples treated at high temperature during GS3 showed alteration of the concentrations of nine metabolites: 2-oxoglutaric acid, beta-alanine, galacturonic acid, gamma-aminobutyric acid (GABA), guanosine, methionine sulfoxide, phenylalanine and proline increased, whereas sinapic acid decreased. In GS1–3, only kynurenic acid decreased compared with the control. No metabolites were observed in the samples treated at high temperature only during GS1.

Sixteen metabolites changed relative to the control in two or more conditions: Citrulline changed in all four treatments (decreased in GS3 and GS1–3, and increased in GS1 and GS2). Arginosuccinic acid and glutamic acid altered in GS2, GS3, and GS1–3, with increase in GS2. Ascorbic acid decreased by similar amounts in GS2, GS3, and GS1–3. Putrescine decreased in GS2, GS3, and GS1–3, with the greatest decrease in GS1–3. Tartaric acid increased by comparable amounts in GS2, GS3, and GS1–3. Pyroglutamic acid increased in GS1, GS2, and GS1–3, with the greatest increase in GS2. Cytosine increased in GS2 and GS1–3, with the greatest increase in GS2. Histidine increased by similar amounts in GS2 and GS3. Hypoxanthine and inosine decreased by comparable amounts in GS1 and GS1–3. NAD increased in GS1 and GS2, and decreased in GS3. Serotonin and tryptophan increased in GS3 and GS1–3, with the greatest increase in GS3. Succinic acid increased by comparable amounts in GS2 and GS1–3.

GS2 and GS3 plants accumulated free amino acids but with different trends. Of the 27 amino acids, 22 showed changes relative to the control under one or more conditions (Table 1). Sixteen were altered by the GS2 treatment, and all except hydroxyproline increased. Only histidine showed the same increase in GS2 and GS3, but three amino acids (argininosuccinic acid, citrulline, and glutamic acid) responded differently between GS2 and GS3 plants. The remaining six amino acids (beta-alanine, GABA, methionine sulfoxide, phenylalanine, proline, and tryptophan) increased by the treatment in GS3. The GS1–3 plants responded differently from the GS2 and GS3 plants, and were not significantly different from the control, except for four amino acids (argininosuccinic acid, citrulline, glutamic acid, and tryptophan), which showed similar behavior to that in the GS3 plants. Pyroglutamic acid showed more than two times the control value in all treatments except GS3.

### 2.4. Enrichment Analysis of Differentially Accumulated Metabolites Corresponding to Stage-Specific Heat Stress

To understand how high temperature at different growth stages altered metabolic pathways during the reproductive stage, we conducted enrichment analysis using differentially accumulated metabolites (DAMs), and mapped the metabolites showing interesting behavior in the associated metabolic pathways (Table S3, Figure 4 and Figure 5).

The urea cycle was commonly affected in GS1, GS2, and GS3, but not in GS1–3. Eight metabolites (argininosuccinic acid, fumaric acid, glutamic acid, aspartic acid, 2-oxoglutaric acid, arginine, NAD, and citrulline) were included in the urea cycle. Both in GS2 and GS3, arginine and proline metabolism (glutamic acid, proline, argininosuccinic acid, glycine, fumaric acid, aspartic acid, succinic acid, arginine, NAD, and citrulline), aspartate metabolism (argininosuccinic acid, beta-alanine, fumaric acid, glutamic acid, asparagine, aspartic acid, 2-oxoglutaric acid, arginine, and citrulline), the malate–aspartate shuttle (glutamic acid, aspartic acid, and malic acid), and ammonia recycling (glycine, glutamic acid, asparagine, histidine, aspartic acid, 2-oxoglutaric acid, and NAD) were detected (Table S3). Some pathways reacted only in a specific treatment group: purine metabolism in GS1 (fumaric acid, hypoxanthine, inosine, and NAD), carnitine synthesis (ascorbic acid, glycine, succinic acid, and NAD), and tyrosine metabolism in GS2 (ascorbic acid, fumaric acid, glutamic acid, tyrosine, aspartic acid, tyramine, and NAD). In GS3, metabolites involved in beta-alanine metabolism (beta-alanine, glutamic acid, histidine, 2-oxoglutaric acid, and NAD), the glucose–alanine cycle (glutamic acid, 2-oxoglutaric acid, and NAD), and tryptophan metabolism (glutamic acid, 2-oxoglutaric acid, serotonin, NAD, and tryptophan) changed significantly.

### 2.5. Correlation with the Altered Metabolite Concentrations and Agronomic Traits

**Figure 6:**
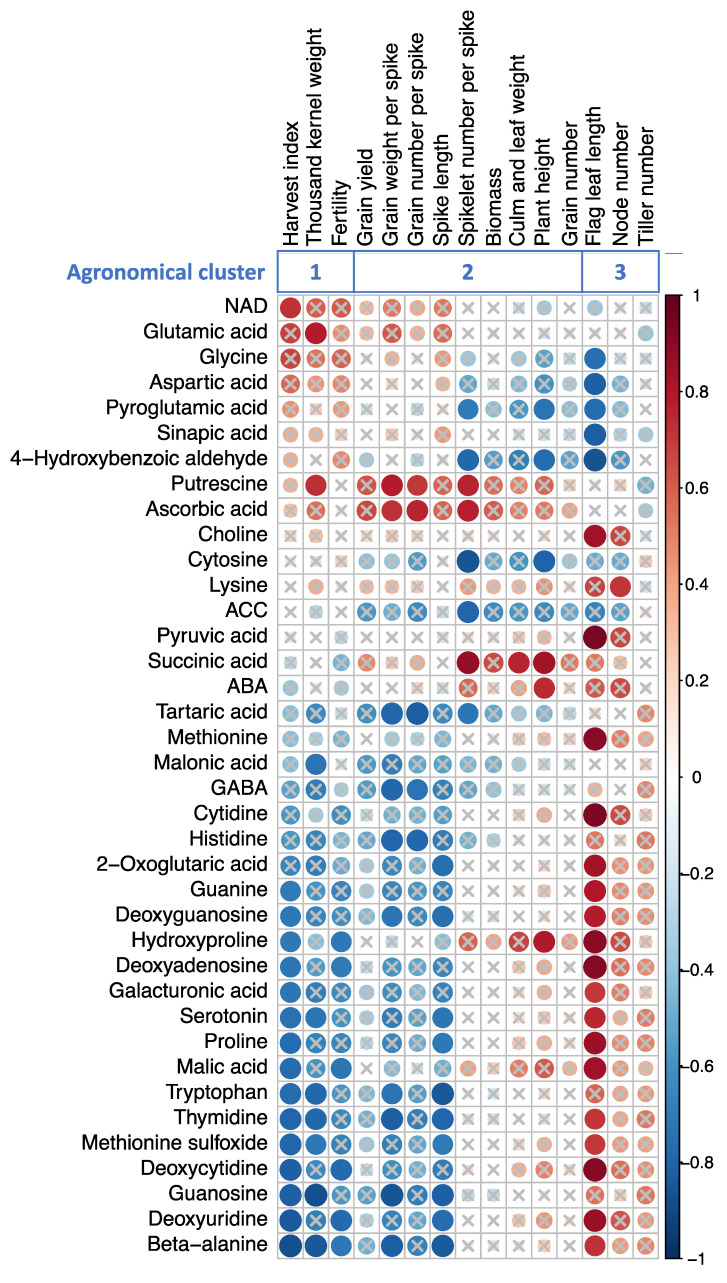
Heatmap of a correlation matrix of agricultural traits and metabolites. Pearson correlation coefficients were calculated using data of all growing conditions. The metabolites that have correlation coefficients greater than 0.7 with at least one agronomical trait are shown. Color and size of circles indicate value of coefficients (red for positive and blue for negative). Crosses are drawn above circles where absolute values of coefficients are smaller than 0.7 and significant after the Benjamini-Hochberg correction. Abbreviations: NAD, nicotinamide adenine dinucleotide; ACC, 1-aminocyclopropane-1-carboxylic acid; ABA, abscisic acid; GABA, gamma-aminobutyric acid.

The changes in metabolite concentrations described in the previous sections were correlated with the values of the agronomic traits obtained in our previous study (Figure 6, Tables S4 and S5) [12]. Matsunaga et al. [12] divided agronomic traits into three clusters based on the response to high-temperature treatment: in agronomical cluster 1, traits were positively affected by heat during GS1; in agronomical cluster 2, traits were negatively affected by heat during all stages; and in agronomical cluster 3, traits were affected negatively by heat during GS1 but positively during GS3.

Many metabolites in the present study showed strong correlations with the agronomic traits classified in agronomical cluster 1 (harvest index, thousand-kernel weight, and fertility). These metabolites were negatively correlated with the harvest index: methionine sulfoxide, proline, beta-alanine, tryptophan, guanosine, deoxyguanosine, thymidine, guanine, deoxyuridine, deoxycytidine, deoxyadenosine, galacturonic acid, malic acid, hydroxyproline, and serotonin, and only NAD showed a positive correlation with this trait. In contrast, putrescine and glutamic acid were positively correlated with the thousand-kernel weight, whereas methionine sulfoxide, beta-alanine, tryptophan, guanosine, thymidine, malonic acid, and serotonin were negatively correlated. Beta-alanine, deoxyuridine, deoxycytidine, deoxyadenosine, and hydroxyproline were negatively correlated with fertility.

Many metabolites showed strong correlations (with the agronomic traits classified in agronomical cluster 2 (grain weight per spike, grain number per spike, spike length, spikelet number per spike, culm and leaf weight, and plant height)). For grain weight per spike, putrescine and ascorbic acid were positively correlated, whereas histidine, beta-alanine, GABA, tryptophan, guanosine, deoxyguanosine, thymidine, and tartaric acid were negatively correlated. For grain number per spike, putrescine and ascorbic acid showed positive correlation, whereas histidine, GABA, and tartaric acid showed negative correlations. For spike length, methionine sulfoxide, proline, beta-alanine, tryptophan, guanosine, deoxyguanosine, thymidine, deoxyuridine, 2-oxoglutaric acid and serotonin showed negative correlations, and no metabolites showed a positive correlation. For spikelet number per spike, putrescine, ascorbic acid, and succinic acid showed positive correlations, whereas cytosine, pyroglutamic acid, tartaric acid, 4-hydroxybenzoic aldehyde, and 1-aminocyclopropane-1-carboxylic acid (ACC) showed negative correlations. For culm and leaf weight, succinic acid showed a positive correlation and there were no negative correlations. For plant height, succinic acid, ABA, and hydroxyproline showed positive correlations, whereas cytosine, pyroglutamic acid, and 4-hydroxybenzoic aldehyde showed negative correlations.

In the agronomic traits classified as agronomical cluster 3 (flag leaf length and node number), many metabolites showed strong correlations: aspartic acid, glycine, pyro-glutamic acid, 4-hydroxybenzoic acid, and sinapic acid were negatively correlated with flag leaf length, whereas many metabolites were positively correlated with this trait: methionine sulfoxide, proline, beta-alanine, choline, methionine, deoxyguanosine, thymidine, guanine, cytidine, deoxyuridine, deoxycytidine, deoxyadenosine, galacturonic acid, malic acid, pyruvic acid, 2-oxoglutaric acid, hydroxyproline, and serotonin. For node number, only lysine showed a correlation, and it was positive.

We found no metabolites that were significantly correlated with grain yield, tiller number, biomass, and grain number.

## 3. Discussion

### 3.1. Metabolites Involved in Senescence Can Explain Heat Priming and Acclimation

Matsunaga et al. [12] reported that control plants grown under standard conditions showed the onset of leaf senescence at 14 DPA and that the plants treated with high temperature during GS3 showed apparent leaf senescence by 14 DPA after losing carbon assimilation capacity. On the other hand, plants treated with a high temperature during all growth stages (GS1–3) were able to maintain high photosynthetic activity even at 14 DPA. This suggests that high-temperature exposure during the seedling stage (GS1), vegetative stage (GS2), or both induced high-temperature acclimation during the seed maturation stage (GS3). Moreover, plants treated with high temperature only in GS1 showed higher photosynthetic activity than the control. Thus, we regarded treatment during GS1 as a “priming effect” that delayed senescence during the seed maturation stage.

**Figure 7:**
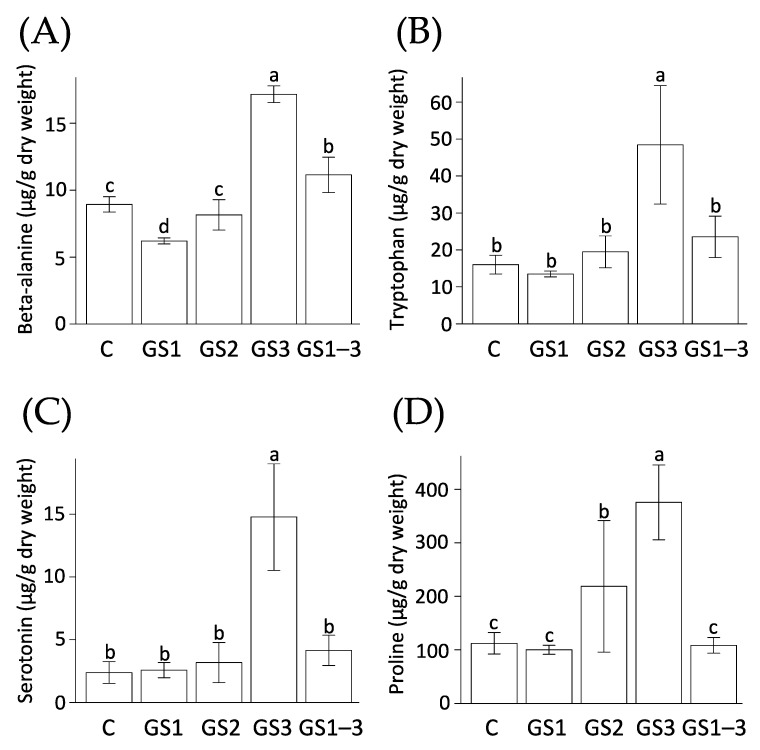
Contents of (**A**) beta-alanine, (**B**) tryptophan, (**C**) serotonin, and (**D**) proline. Values are means ± SD (*n ≥* 2). Growth stages: C = control, with normal temperatures; GS1 = heating from germination to tillering; GS2 = heating from tillering to flowering; GS3 = heating from flowering to full maturity, and GS1–3 = heating during all growth stages. Values of a given metabolite labeled with the same letter did not differ significantly (Tukey’s range test, *p* < 0.05). The data labeled 2019 and 2020 represents samples grown in different years.

In the present study, we investigated the metabolomic dynamics behind this phenomenon. The GS3 plants showed alterations of levels of metabolites involved in beta-alanine synthesis (Figure 4), and accumulated more beta-alanine (Figure 7A). However, its accumulation in GS1–3 plants was significantly lower than that in GS3 plants. Moreover, the amount of beta-alanine in GS1 plants was significantly lower than that in the control. In the DNA and RNA degradation pathways, nucleotidases catalyze the transformation of nucleotides into nucleosides and phosphates, followed by nucleosidase activity to generate purines and pyrimidines [14,15]. Generally, purines and pyrimidines are either regenerated into nucleic acids through salvage and phosphorylation, or are degraded into amino acids, urea, ribose, NH_4_^+^, and CO_2_ under nitrogen starvation and during leaf senescence. Matsunaga et al. [12] measured that the rate of carbon assimilation at 14 DPA by photosynthesis measurement and found decrease in the order GS3 > GS1–3 > GS2 > control > GS1. In the present study, we found that the accumulation of beta-alanine at 7 DPA showed the same trend (Figure 7A). Beta-alanine results from degradation of uracil produced during pyrimidine degradation by dihydropyrimidine dehydrogenase, dihydropyrimidinase, and beta-ureidopropionase. Therefore, the higher accumulation of beta-alanine during GS3 may indicate that nucleotide degradation leading to pyrimidine degradation under heat stress has already occurred. This is consistent with the findings of Avila-Ospina et al. [16], who reported that beta-alanine accumulates in the flag leaves of barley during leaf senescence. Therefore, beta-alanine accumulation can be a biomarker for the degree of senescence.

Similar to the response of pyrimidine catabolism to heat stress, the accumulation of hypoxanthine in GS1 and GS1–3 plants was remarkably lower than in the control (Table 1). Hypoxanthine, which is generated from inosine by inosine nucleosidase, represents a turning point in the salvage pathway of this metabolite for regeneration of purine nucleotides or degradation of purine [15]. Production of the enzyme xanthine dehydrogenase/oxidase (XDH), which is responsible for uric acid synthesis from hypoxanthine, is up-regulated in response to ABA [17]. Inhibition of XDH may play an essential role in the maintenance of plant growth and development by inducing various effects such as growth retardation, reduced fertility, and premature maturation [17,18,19]. The low accumulation of hypoxanthine in GS1 and GS1–3 plants may therefore result from increasing activity of XDH in purine degradation. It may also be related to the delayed senescence observed in GS1 plants compared to the control and in GS1–3 plants compared to GS3 plants.

Alteration of metabolite levels related to the amino acids also appeared in addition to the nucleic acid pathways mentioned above. Plants exposed to high temperature during the seedling stage (GS1) accumulated the least tryptophan and serotonin (Figure 7B,D) in the tryptophan metabolic pathway. In contrast, plants exposed to high temperatures at the sampling time (GS3 and GS1–3) had higher levels of both metabolites, and the difference was significant in the case of the GS3 plants. The accumulation of these amino acids in GS3 plants was more than twice that in GS1–3 plants. The trend of these accumulations is similar to the senescence levels at 14 DPA [12].

The tryptophan metabolic pathway diverges at tryptamine and is divided into the synthesis of indole-3-acetic acid via indole-3-pyruvic acid and the synthesis of serotonin [20]. Tryptamine biosynthesis involves tryptophan decarboxylase, whose gene expression increases during senescence and is thought to be the rate-limiting factor for serotonin accumulation [21]. Serotonin plays a protective role against reactive oxygen species in plants, and by regulating cell division and elongation, it delays senescence [22]. Furthermore, Ishihara et al. [23] reported that serotonin is involved in establishing a physical defense against pathogens in rice. However, this does not mean that plants with a high accumulation of serotonin exhibit delayed senescence, but rather that senescent plants increase their serotonin levels to resist the effects of senescence. In any case, the serotonin accumulation (Figure 7C) could be an indicator of the degree of senescence.

We observed similar behavior for proline, whose accumulation was lowest in GS1 plants and highest in GS3 plants (Figure 7D). Yuan et al. [4] reported that photosynthetic activity was reduced by high temperature in *Brassica napus*, and that proline showed the opposite accumulation tendency to that of several amino acids, and explicitly increased under stress. Proline is known to be a compatible solute that increases cell osmotic pressure under drought stress [3]. However, the role of proline in high-temperature stress is evident but not yet fully understood [24].

Our results indicated that the amino acids beta-alanine, tryptophan, serotonin, and proline will be useful molecular markers for indicating senescence.

### 3.2. Metabolites Involved in Ammonia Recycling and Urea Cycle Indicate Recovery from Heat Stress

Our study demonstrated that high temperatures during the vegetative stage (GS2) and reproductive stage (GS3) led to accumulation of different amino acids. Plants exposed to high temperatures during GS3 showed increased beta-alanine, tryptophan, serotonin, and proline levels, as described in the previous section. On the other hand, those exposed to high temperature during GS2 accumulated other amino acids (e.g., leucine, isoleucine, threonine, tyrosine, and valine). The increase in these amino acids may be due to a stress-induced decrease in protein synthesis or increase in protein degradation [5]. The amino acids that increased in GS3 plants were those that accumulate during senescence, whereas the amino acids that increased in GS2 plants were those that remain after the effects of high temperature before flowering. NH_4_^+^, as a protein degradation product, is consumed by glutamic acid dehydrogenase (GDH) and glutamine synthase (GS) and in the urea cycle (Figure 4). Plants exposed to high temperature during GS2 accumulated significantly more arginine, argininosuccinic acid, and citrulline, which are three of the four major metabolites in the urea cycle (the fourth is ornithine), indicating that detoxification of NH_4_^+^ by conversion into urea in this metabolic pathway is particularly active in the GS2 plants. In the urea cycle, NH_4_^+^ is consumed during the synthesis of citrulline from ornithine. Therefore, the metabolic activity of ornithine conversion to citrulline may be accelerated in the GS2 plants and NH_4_^+^ consumption may be increased.

Blume et al. [25] reported that ornithine and citrulline serve as alternative sinks for excess nitrogen, and that these may be also alternative sinks for nitrogen produced during photorespiration. NH_4_^+^ produced by protein degradation accumulates in metabolites in the form of nitrogen. Arginine, which functions in part of the urea cycle, functions as a major nitrogen storage compound in plants [25]. The observed increase in arginine and argininosuccinic acid in the GS2 plants in the present study suggests that nitrogen accumulation is occurring in these plants at 7 DPA. However, among the four metabolites involved in the urea cycle, the content of argininosuccinic acid was by far the highest, suggesting that nitrogen accumulation is likely to occur in this acid rather than in arginine.

Other ammonium recycling pathways include the GDH pathway and the GS–glutamine oxoglutarate aminotransferase (GOGAT) cycle (Figure 4). In the GDH pathway, NH_4_^+^ is consumed by the catalytic action of GDH during the synthesis of glutamate from 2-oxoglutarate in the tricarboxylic acid cycle. In the GS–GOGAT cycle, NH_4_^+^ is consumed by GS during the synthesis of glutamine from glutamate, and NH_4_^+^ is released from glutamine during the synthesis of glutamic acid catalyzed by glutamine oxoglutarate aminotransferase. In the present study, glutamate accumulated in plants exposed to high temperature during GS2, but accumulated less in plants treated with high temperature during the reproductive stage (GS3 and GS1–3). The reduced accumulation of glutamic acid in GS3 plants may be due to increased GDH activity and decreased GS–GOGAT activity caused by exposure to high temperature during the reproductive stage. Yuan et al. [4] and Blume et al. [26] reported that high temperature-sensitive wheat lines showed a decrease in GS–GOGAT activity and an increase in GDH activity under high temperature, whereas resistant lines showed a smaller reduction.

Furthermore, glutamic acid is converted to aspartic acid by aspartic acid aminotransferase, and aspartic acid is converted to asparagine by asparagine synthase and asparaginase [23] (Figure 4). Glutamine, glutamic acid, and aspartic acid are used in the synthesis of nitrogenous compounds. Asparagine is synthesized from aspartic acid, and functions not only as a protein amino acid but also as a major nitrogen transporter [27]. In the present study, aspartic acid, asparagine, and glutamate accumulated in plants exposed to high temperature during GS2. This suggests that nitrogen assimilation, metabolism, and transport are activated by high-temperature exposure during GS2. In addition, GS2 plants may be preparing to resume protein synthesis at 7 DPA, and accumulated nitrogen stores in asparagine and arginine would be the materials consumed by this process.

In healthy plants, when photosynthesis is active during the daytime, glutamine, glutamic acid, and aspartic acid are used to synthesize other amino acids required for protein synthesis. These are converted to asparagine at night, which functions as a nitrogen storage and transport compound [28]. On the other hand, in carbon-starved plants, glutamine and glutamic acid are converted to sugars, and the nitrogen released in this process is stored in nitrogen-rich metabolites such as asparagine and arginine [29]. In this study, plants exposed to high temperatures during GS2 accumulated particularly high levels of asparagine and arginine, suggesting that these plants are carbon-starved. In carbon-starved plants where the stomata are closed and CO_2_ is not supplied, the CO_2_ concentration in the leaves decreases significantly, resulting in increased photorespiration [30]. Photorespiration functions to prevent the accumulation of reactive oxygen species due to excessive photosynthetic light reactions [31].

Accumulation of glycine, serine and glyceric acid are reported to be conversion from the toxic compound, 2-phosphoglycolate by photorespiration [32]. Thus, plants exposed to high temperatures during GS2 may cause increase of photorespiration at 7 DPA as a post-heat stress feature. The NH_4_^+^ emitted by photorespiration is assimilated by GS in the GS–GOGAT cycle [30]. In addition, since nitrogen assimilation, metabolism, and transport appear to be activated in GS2 plants, the mechanism for removing the NH_4_^+^ generated by photorespiration may also be activated.

These facts indicate that the impact of high temperature in GS2 plants continued into the GS3 stage even though the plants grew 7 days under control conditions. These plants are recovering from mitigating the adverse effects of temperature stress by actively performing nitrogen metabolism and photorespiration to remove NH_4_^+^ and reactive oxygen species.

### 3.3. Metabolites Can Explain Agronomic Traits Alteration by Heat Stress

Many of the metabolites were strongly correlated with agronomic traits. In particular, four metabolites (beta-alanine, serotonin, tryptophan, and proline) showed strong negative correlations with harvest index and spike length, and a strong positive correlation with flag leaf length. Beta-alanine, serotonin, and tryptophan were also negative correlated with thousand-kernel weight and grain weight per spike, and beta-alanine showed a negative correlation with fertility. These metabolites were regarded as indicators of senescence. The results suggest that senescence affects grain formation. In addition to plant senescence, starch accumulation is another factor that affect grain formation. Lu et al. [33] and Yang et al. [34] reported that heat stress during grain filling stage affects the activities of starch biosynthesis genes responsible for enzymes such as sucrose synthase, AGPase, glucokinase, soluble starch synthase, and starch branching enzyme that are involved in starch accumulation in wheat and maize. Additionally, at 7 DPA, the GS3 heat treatment at 38 °C/18 °C reached 200 cumulative degree-days of heat, indicating that 7 DPA in GS3 overlaps the timing of A-type starch granule synthesis [35,36]. Thus, translocation of nutrients from senescing leaf tissue accelerated at 7 DPA, and may have led to the highest fresh weight accumulation in the grains at 10 DPA in GS3 plants even though the grain dry weight did not increase significantly [12]. This suggests a possible defect of starch synthesis in hot environments rather than a problem with nutrient translocation from the source (senescing tissue). It is possible that less-effective starch biosynthesis during grain development under heat stress may lead to use of sugars and nutrients for flag leaf growth, therefore leading to increased flag leaf length.

As for the other metabolites, putrescine showed a strong positive correlation with four agronomic traits: thousand-kernel weight, grain weight per spike, grain number per spike, and spikelet number per spike (Figure 6). The accumulation of putrescine was significantly less in all plants that experienced high temperatures than in the control, and the order was GS1 > GS2 > GS3 > GS1–3.

Putrescine is the major polyamine in plants and is involved in the regulation of a variety of physiological processes, including senescence and embryogenesis [37]. Polyamine metabolic enzyme activities and polyamine content change throughout a plant’s growth stages. Duan et al. [38] and Duan [39] reported that this senescence could be inhibited by the application of exogenous polyamines, and indicated that this change in the polyamine content could be a signal of or prelude to senescence. Polyamines are regulators of embryonic development. Putrescine is abundant in immature embryos, and reduced concentrations of putrescine would therefore result in fewer somatic embryos. Few reports have mentioned the relationship between high-temperature stress and polyamines. Shao et al. [40] reported that the heat tolerance of alfalfa is associated with a high spermidine content and low putrescine and spermine contents. A high-temperature treatment also affected polyamine synthesis in Chinese kale leaves, and the total amount of polyamines and the content of putrescine increased after 6 days of high-temperature treatment, but the increase was not sustained as the treatment time increased [41]. These results suggest that putrescine is also useful as an indicator of senescence and indirectly explains the status of embryogenesis at 7 DPA. However, there have been no reports that could fully explain the high temperature priming effect and the behavior of high temperature acclimation seen in GS1–3 plants in the present study. Although beta-alanine (the end product of polyamine synthesis [42]) and putrescine were significantly correlated, the correlation coefficient was −0.52, indicating that there was a weak or indirect relationship between them.

The correlations with agronomic traits indicates that the behavior of these metabolites at 7 DPA influences subsequent seed development, suggesting that these substances are essential as the basis for the expressed traits. We hypothesize that metabolites analysis can be used to estimate yield in the future.

## 4. Materials and Methods

### 4.1. Plant Material and Growth Conditions

We grew the bread wheat (*Triticum aestivum* L.) cultivar ’Norin 61’ under heat treatment during specific growth stages and measured 15 agronomic characteristics (harvest index; thousand-kernel weight; fertility; grain yield; grain weight per spike; grain number per spike; spike length; spikelet number per spike; biomass; culm and leaf weight; plant height; grain number; flag leaf length; node number; tiller number) following the methods of Matsunaga et al. [12]. In summary, plants were grown in climate chambers in which temperature, humidity, and light were controlled as below (Espec, Osaka, Japan: W 1800 mm × D 1800 mm × H 2500 mm) at the Arid Land Research Center, Tottori University, Japan. Owing to limited space in the chambers, plants were grown in two seasons (March–July 2019 and January–May 2020). In each experiment, one planter (46.5 cm × 23.7 cm × 17.5 cm) with six plants was used for each heat treatment (38 °C). We designated three growth stages: GS1 (Zadoks’s scale, Z12 to Z19), from germination to tillering; GS2 (Z20 to Z61), from tillering to flowering; and GS3 (Z62 to Z95), from flowering to full maturity [43,44] (Figure 1). We also applied the heat treatment throughout plant growth (GS1–3). The results for the agronomic and physiological traits, and for photosynthesis and seed characteristics in these experiments, were recently published [12].

### 4.2. Metabolite Analysis

We collected six flag leaves from two plants (three from each plant) at 7 DPA, and flash-frozen these samples in liquid nitrogen. We pulverized the samples using a Multi-Beads Shocker (Yasui Kikai, Osaka, Japan) without thawing, and then stored the samples at −80 °C. We dehydrated the powder using a VD-550R freeze-dryer (TITEC, Saitama, Japan). We then extracted 4 mg of the dry powdered sample with 400 μL of 80% *v*/*v* MeOH (LC/MS grade; FUJIFILM Wako Chemicals, Osaka, Japan; cat# 138-14521) overnight at room temperature in the dark.

After centrifugation (15,000 rpm, 5 min, 4 °C), the supernatant was collected, and then centrifuged again to remove any insoluble material. In the supernatant, we quantified the concentrations of 69 metabolites (Table S1) using a triple-quadrupole LC-MS/MS system (Agilent 6420, Santa Clara, CA, USA) and a Discovery HS-F5 column (2.1 × 250 mm, 5 μm i.d.; Sigma-Aldrich, Allentown, PA, USA). Metabolites were identified by means of multiple reaction monitoring analysis. The product ions used to characterize each metabolite are shown in Supplementary Materials Table S1.

We standardized the metabolite levels in each sample per unit sample dry weight, and then calculated *z*-scores relative to the control value. Standard curves for the metabolites were established using laboratory standard solutions at different concentrations (0.0, 0.4, 2.0, and 10.0 ppm). Compounds with similar molecular weights or retention times were not included in the same mixture. The mobile phase consisted of 0.1% *v*/*v* formic acid (LC/MS grade; FUJIFILM Wako Chemicals, Osaka, Japan; cat# 067-04531) and acetonitrile (LC/MS grade; FUJIFILM Wako Chemicals, Osaka, Japan; cat# 018-19853) as the A and B solutions, respectively. We then applied four A:B ratio gradient flows: (1) 100% A: 0% B for 2 min, (2) 75% A: 25% B for 8 min, (3) 65% A: 35% B for 4 min, and (4) 5% A: 95% B for 3 min. We evaluated the peaks derived from the metabolites with a high signal-to-noise ratio to exclude unreliable peaks. Quantifications for each metabolite between replications were validated with relative standard deviation (RSD) less than 30%. The metabolite concentrations obtained from each condition was divided by the mean value of the control, and the fold change from the control was calculated. The values were used for enrichment analysis and multivariate analysis.

### 4.3. Statistical Analysis

We analyzed the dataset using MetaboAnalyst 5.0 (https://www.metaboanalyst.ca/ accessed on 15 October 2021), which is Web-based metabolomics data analysis software. We excluded metabolites with missing values in even one replication under each condition, and performed our analyses using only metabolites that were stably detected in all replicates with RSD less than 30%. One of the three iterations of GS2 showed clear outliers for all metabolites, so we excluded it from the analysis.

We applied univariate analysis to calculate the change in metabolite concentrations between each heat-stressed condition and the control. To assess the statistical significance of our results, we used *t*-tests and one-way ANOVA using R version 4.1.0 (https://www.R-project.org/ accessed on 15 October 2021). *p* values less than 0.05 were considered statistically significant. We applied the multivariate method principal component analysis to provide additional insights into the relationships among the variables. For the metabolites that showed a significant change (*p* < 0.05), we performed pathway analysis and enrichment analysis using MetaboAnalyst 5.0. The enrichment analysis selected the metabolic pathways with false discovery rate (FDR) less than 0.05.

Pearson correlation coefficients were used to evaluate relationships between metabolites and agricultural traits. We tested whether the correlation coefficients were significantly different from 0 (*p* < 0.05). The Benjamini-Hochberg correction was applied to adjust the significance level in a multiple test. The values of the abundance of 69 metabolites and 15 agronomic traits of the corresponding samples were used in the analysis.

## 5. Conclusions

We explained the priming effect caused by high temperature, accelerated senescence, and high-temperature acclimation based on the dynamics of a large number of metabolites. Beta-alanine, tryptophan, serotonin, proline, and putrescine in the flag leaves at 7 DPA all appear to be suitable biomarkers that can be used to evaluate senescence and acclimation. In particular, accumulation of beta-alanine in the flag leaves could indicate level of leaf senescence in response to heat stress, while increase in tryptophan and serotonin appeared as ongoing heat stress markers but disappeared in the acclimated sample. These metabolites were strongly correlated with agronomic traits such as the thousand-kernel weight. We also observed changes in metabolite concentrations during the recovery from the damage caused by heat stress.

## Figures and Tables

**Table 1:** Changes compared with the control for all metabolites in the heat-stressed plants.

Metabolites	GS1	GS2	GS3	GS 1–3	Metabolites	GS1	GS2	GS3	GS 1–3
ABA	0.92	**0.63**	1.09	0.76	Kynurenic acid	0.78	0.98	1.01	**0.63**
ACC	0.84	1.36	1.07	1.38	Leucine *	0.92	**1.78**	0.93	0.95
Adenosine	0.88	1.12	1.13	0.97	Lysine *	1.02	1.03	1.00	0.85
Alanine *	0.91	1.39	1.36	1.14	Malic acid	0.77	**0.49**	1.22	0.95
Arginine *	1.16	**1.61**	1.17	1.01	Malonic acid	1.19	1.01	1.30	1.33
Argininosuccinic acid *	1.42	**3.26**	**0.55**	**0.52**	Methionine *	1.08	1.20	1.40	0.91
Ascorbic acid	1.04	**0.48**	**0.59**	**0.60**	Methionine sulfoxide *	0.68	0.73	**1.89**	1.16
Asparagine *	0.97	**3.78**	0.96	1.00	NAD	**2.07**	**2.30**	**0.57**	0.75
Aspartic acid *	1.10	**1.93**	0.76	1.10	Nicotinic acid	0.82	1.10	0.98	0.86
Beta-alanine *	0.70	0.93	**1.90**	1.23	Ornithine *	1.12	0.87	0.97	0.77
Betaine	1.00	**3.00**	1.22	1.31	2-Oxoglutaric acid	1.18	1.25	**2.01**	1.11
Choline	1.12	1.13	1.06	0.89	Phenylalanine *	0.96	1.26	**1.53**	1.06
Citric acid	0.93	**0.60**	1.16	1.30	Proline *	0.80	1.12	**3.78**	1.09
Citrulline *	**1.66**	**3.45**	**0.37**	**0.28**	Putrescine	0.77	**0.59**	**0.41**	**0.26**
Cytidine	0.86	1.11	1.22	0.90	4-Pyridoxic acid	1.15	1.25	1.00	1.09
Cytosine	1.08	**2.14**	1.27	**1.70**	Pyroglutamic acid *	**2.25**	**8.55**	0.98	**3.15**
Deoxyadenosine	0.83	1.03	1.27	0.95	Pyruvic acid	1.04	1.21	1.20	0.83
Deoxycytidine	0.78	0.92	1.37	0.94	Salicylic acid	0.98	1.36	0.70	0.89
Deoxyguanosine	0.82	1.13	1.46	1.06	Serine *	1.20	**3.10**	0.79	0.81
Deoxyuridine	0.79	0.90	1.32	0.97	Serotonin	0.84	1.36	**8.69**	**2.43**
Fumaric acid	**0.65**	**0.46**	1.40	1.37	Shikimic acid	1.19	**0.25**	1.07	0.69
GABA *	0.97	1.39	**1.66**	1.44	Sinapic acid	0.80	0.77	**0.43**	1.08
Galacturonic acid	1.00	0.82	**1.71**	1.10	Succinic acid	0.75	**0.35**	0.85	**0.56**
Glutamic acid *	1.06	1.53	**0.63**	**0.65**	Tartaric acid	0.80	**2.24**	**2.14**	**1.99**
Glutamine *	1.00	1.06	0.96	0.82	Threonine *	1.10	**1.87**	0.90	0.87
Glyceric acid	1.02	1.45	0.78	0.82	Thymidine	0.87	1.05	1.49	1.14
Glycine *	1.33	**1.88**	0.76	1.02	Trigonelline	0.85	**0.59**	0.88	0.72
Guanine	0.76	1.05	1.38	1.00	Tryptophan *	0.79	0.98	**3.23**	**1.57**
Guanosine	0.88	0.99	**1.60**	1.29	Tyramine	1.22	**1.56**	1.19	1.06
Histidine *	0.84	**1.54**	**1.78**	1.37	Tyrosine *	1.01	**1.87**	1.27	1.11
4-Hydroxybenzaldehyde	0.95	1.42	0.89	1.29	Uridine	0.81	1.09	1.14	0.92
Hydroxyproline *	0.68	0.39	1.28	0.68	Valine *	1.06	**2.39**	0.89	0.86
Hypoxanthine	**0.60**	**1.39**	0.79	**0.62**	Vanillin	0.85	0.73	0.84	0.90
Inosine	**0.61**	1.25	0.72	**0.66**	Xanthosine	1.04	1.35	0.83	1.00
Isoleucine *	0.93	**2.39**	1.04	0.94					

Note: Growth stages: GS1 = heating from germination to tillering; GS2 = heating from tillering to flowering; GS3 = heating from flowering to full maturity, and GS1–3 = heating from germination to full maturity. Values that represent a significant increase (more than one and a half fold) or decrease (less than two-thirds) the control values are shown in bold. Metabolites labeled with an asterisk are amino acids. Abbreviations: ABA, abscisic acid; ACC, 1-Aminocyclopropane-1-carboxylic acid; GABA, gamma-aminobutyric acid; NAD, nicotinamide adenine dinucleotide.

## Data Availability

Not applicable.

## Supplementary Materials

The following are available online at https://www.mdpi.com/article/10.3390/ijms222313139/s1.
